# Effect of transition metal (TM) doping on structural and magnetic properties in hexagonal YMn_0.917_TM_0.083_O_3_ systems

**DOI:** 10.1016/j.heliyon.2018.e00993

**Published:** 2018-12-08

**Authors:** Dong Chen, Yu-Jia Wang, Yin-Lian Zhu, Xiu-Liang Ma

**Affiliations:** Shenyang National Laboratory for Materials Science, Institute of Metal Research, Chinese Academy of Sciences, Wenhua Road 72, 110016 Shenyang, China

**Keywords:** Condensed matter physics

## Abstract

Suitable TM doping at the Mn site is an important access to manipulate magnetic properties of hexagonal YMnO_3_, however, it has not yet been systematically explored how the strength of antiferromagnetic interactions and the magnetic transition temperatures (*T*_N_) are modified in the doping YMn_0.917_TM_0.083_O_3_ systems. In the work, we have performed first-principles calculations to study the effect of TM doping on the structural and magnetic properties of hexagonal YMn_0.917_TM_0.083_O_3_ bulks; the results are combined with the available experimental results. The calculated results reveal that the planar TM-O bonds and O-TM-O angles of TMO_5_ bipyramid are both prominent structural features for the transformations of magnetic properties. We have also predicted the Ti, V, Cr and Fe doping effects on magnetic properties and further analyzed the TM electronic structures of TMO_5_ bipyramid in the YMn_0.917_TM_0.083_O_3_(001)/MgO(001) film configurations, which could provide more understanding towards the designing of new generation multifunctional devices.

## 1 Introduction

Multiferroic materials exhibit simultaneous ferroic responses with coupled electric, magnetic, and structural orders. They are very important because of their unique and strong coupling of electric and magnetic properties, giving rise to the simultaneous presence of more than one ferroic property. Hexagonal YMnO_3_ is the most extensively studied multiferroic material among all hexagonal manganites [1], which possesses magnetism and ferroelectricity simultaneously with a high ferroelectric transition temperature (∼900 K) and a low antiferromagnetic (AFM) transition temperature (∼70 K). The crystal structure of YMnO_3_ (*P*6_3_*cm*) can be described as stacks of corner-linked MnO_5_ trigonal bipyramid (Mn atoms with two apical oxygen (O1 and O2) atoms and three planar oxygen (O3 and O4) atoms) layers separated by Y atom layers along the crystallographic *c*-axis. In the hexagonal structure there appears to be a small tilting (a cooperative tilting distortion which buckles the trigonal planes) of MnO_5_ bipyramids, which consist of five Mn–O bonds: Mn–O1 and Mn–O2 oriented along the *c* direction while Mn–O3 and two pairs of Mn–O4 bonds lie within the *ab* plane. The noncollinear magnetic order is originating from the strong antiferromagnetic superexchange interaction of Mn spins in the *ab* plane of YMnO_3_ structure below the Néel temperature *T*_N_ of 70 K [2, 3]. As the symmetry breaking distortion is driven by geometric but not electronic factors in hexagonal YMnO_3_, there is some freedom to tune the magnetic behavior of manganese atoms by electronic doping, without losing the acentric structure of host phase [4]. Doping atoms with different atomic radius will inevitably change the lattice constant and also the Mn-Mn bond distance. This change in the Mn-Mn bond distance, in particular on the *ab* plane, leads naturally to variations in the exchange integral, which is a critical parameter for the formation of magnetic ground state [5]. Therefore doping at the Mn site in the hexagonal YMnO_3_, one can manipulate the physical properties and change the magnetic ground state.

In recent years, a few studies have been reported on the influence of transition metal (TM) elements doping at Mn-site in hexagonal manganite bulks [5, 6, 7, 8, 9, 10, 11, 12, 13], in which most of doping concentrations were kept about 10% to preserve the phase isostructural. For examples, N. Sharma *et al.* [6] pointed out that doping with 10% Ti at Mn site could reduce the temperature *T*_N_ from 75 K of YMnO_3_ compound to 55 K of polycrystalline YMn_0.9_Ti_0.1_O_3_ synthesized through a solid state reaction. T. C. Han *et al.* [7] found that the antiferromagnetic transition temperature of YMn_1-x_Cr_x_O_3_ (0 ≤ *x* ≤ 0.1) increased from 73 K to 89 K with increasing Cr-content (*x*). And F. Wan *et al.* [8] also reported that the *T*_N_ of YMn_1-x_Cr_x_O_3_ (*x* = 0–0.1) compounds would increase from 73.1 K to 86.1 K. S. Namdeo *et al.* [9] put forward that the Néel temperatures of YMn_1-x_Fe_x_O_3_ (0 ≤ *x* ≤ 0.1) compounds have minutely decreased with increasing Fe- doping concentration (*x*). K. Asokan *et al.* [10] reported that Co, Ni and Cu doping at Mn-site in hexagonal YMnO_3_ with a specific composition of 1/3 would improve the structural and magnetic properties. L. Jeuvrey *et al.* [12] proposed that the magnetic transition temperature *T*_N_ of hexagonal YMn_1-x_Cu_x_O_3_ had decreased from 70 K down to 49 K when *x* went from 0 to 0.15. A. M. Zhang *et al.* [13] revealed that the polycrystalline YMn_1-x_Zn_x_O_3_ with low Zn doping concentration (*x* < 0.1) maintained single phase which demonstrated hexagonal structure with space group of *P*6_3_*cm*, and the *T*_N_ temperatures were respectively 75 K, 65 K and 60 K with *x* = 0, 0.04 and 0.08.

As mentioned above, a systematic understanding of doping effect on *T*_N_ is still lacking although a number of experimental results were given in TM doping YMn_0.9_TM_0.1_O_3_ (TM = Ti∼Zn) bulks. Besides, it remains to be elucidated how the antiferromagnetic interactions and Néel temperatures are modified by dopant incorporation in hexagonal manganite films [14]. In the work, we have firstly used the first-principles method to calculate a variety of TM elements from Ti to Zn atoms doping at the Mn site, and investigate how such doping affects the structural and magnetic properties of hexagonal YMn_0.917_TM_0.083_O_3_ bulks (approximating to experimental YMn_0.9_TM_0.1_O_3_ compositions [5, 6, 7, 8, 9, 10, 11, 12, 13]). Then we have also studied the effect of Ti, V, Cr and Fe doping on magnetic properties and discussed the varied electronic structures of TMO_5_ in the YMn_0.917_TM_0.083_O_3_ film configurations, which could provide more understanding towards the designing of new generation multifunctional devices.

## 2 Calculation

**Fig. 1:**
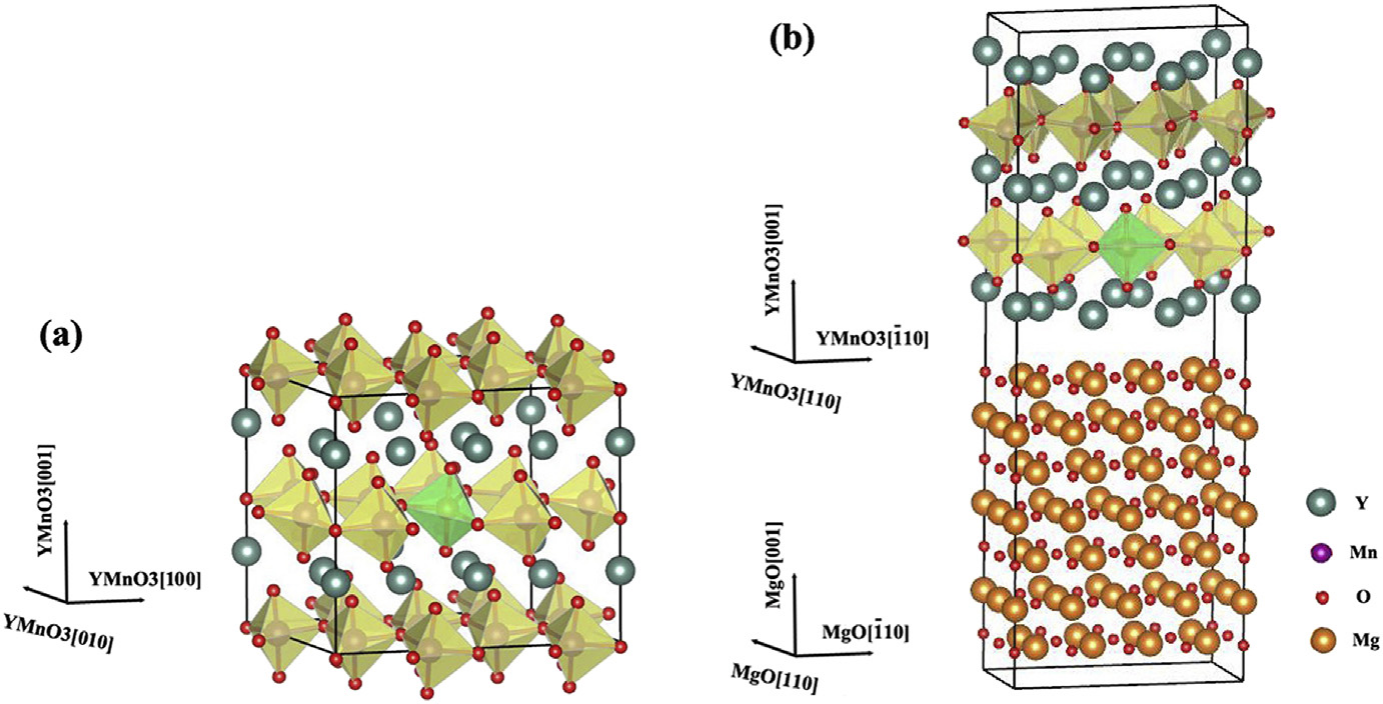
Schematic of the computational cell considered for (a) the hexagonal YMn_0.917_TM_0.083_O_3_ (TM = Ti ∼ Zn) bulk and (b) the YMn_0.917_TM_0.083_O_3_(001)/MgO(001) (TM = Ti, V, Cr and Fe) film configurations, where pine green, purple, red and orange spheres indicate Y, Mn, O and Mg atoms, respectively. On the MnO layer, each Mn atom is surrounded by bipyramidal oxygen atoms which form a MnO_5_ trigonal bipyramid (light yellow). The Mn atom in the green bipyramid will be substituted by a TM atom in the work.

We have performed first-principles density-functional theory (DFT) calculations within the generalized gradient approximation (GGA) [15] using the Vienna *ab initio* Simulation Package (VASP) [16, 17]. The eigenstates of electron wave functions were expanded on a plane-wave basis set using pseudopotentials to describe the electron-ion interactions within the projector augmented-wave approach [18] (PAW). The Monkhorst-Pack (MP) scheme [19] is used for the *k*-point sampling and the Brillouin zone integration is performed with the Gaussian smearing method. The plane-wave basis energy cutoff is chosen as 500 eV. We respectively treated 11 valence electrons for Y (4*s*^2^4*p*^6^5*s*^2^4*d*^1^), 15 for Mn (3*s*^2^3*p*^6^4*s*^2^3*d*^5^), and 6 for oxygen (2*s*^2^2*p*^4^). The atomic positions are fully relaxed until atomic forces are less than 10 meV/Å and the total energy is obtained when it converges to 0.1 meV in the electronic self-consistent loop. In the calculations, the Perdew-Burke-Ernzerhof (PBE) [15] form was chosen as the exchange-correlation potentials within the DFT + U scheme. The DFT+U integrals, determined by the PAW on-site occupancies and the on-site electron-electron interaction, are normally specified in terms of the effective on site Coulomb and exchange parameters, U and J [20]. Here, the values of U = 8.0 eV and J = 0.88 eV are applied for the Mn 3*d* states [21]. U_eff_ is generally expressed as the difference between two parameters (U−J), which determines an orbital-dependent correction to the DFT energy. According to the reported literatures [22, 23, 24, 25, 26, 27, 28, 29], the proper Hubbard correlation item U_eff_ values U_eff_(Ti) = 3.2 eV, U_eff_(V) = 2.64 eV, U_eff_(Cr) = 3.0 eV, U_eff_(Fe) = 4.0 eV, U_eff_(Co) = 6.0 eV, U_eff_(Ni) = 4.7 eV, U_eff_(Cu) = 7.05 eV, U_eff_(Zn) = 8.0 eV are adopted using the Dudarev implementation [30] in the following calculations. To be close to the doping concentration *x* = 0.1 of stoichiometric component YMn_1−*x*_TM_*x*_O_3_ (TM = Ti∼Zn) in the experimental reports [5, 6, 7, 8, 9, 10, 11, 12, 13], we built the supercell of a theoretical formula YMn_0.917_TM_0.083_O_3_ bulk with twelve formula units, in which one of 12 Mn atoms was substituted by a TM atom (shown in Fig. 1(a)). For each computational cell of hexagonal YMn_0.917_TM_0.083_O_3_ (TM = Ti ∼ Zn) bulks (60 atoms) and the YMn_0.917_TM_0.083_O_3_(001)/MgO(001) (TM = Ti, V, Cr and Fe) film [31] configurations (178 atoms) (shown in Fig. 1(b)), we considered three types of magnetic configurations: collinear AFM state, non-collinear magnetic Γ1 state and ferromagnetic (FM) state. It is found that the magnetic configurations have little effects on the optimized lattice constants. During the geometry optimizations, spin-orbit coupling (SOC) and non-collinear magnetism are regarded to calculate the energies of different magnetic configurations.

## 3 Results & discussion

### 3.1 For the hexagonal YMn_0.917_TM_0.083_O_3_ bulks

Firstly we employed the first-principles method to calculate the lattice constants of hexagonal YMn_0.917_TM_0.083_O_3_ (TM = Ti∼Zn) bulks. Table 1 lists the calculated lattice constants for the YMn_0.917_TM_0.083_O_3_ compared to the earlier experimental values of bulk hexagonal YMn_0.9_TM_0.1_O_3_ (TM = Ti∼Zn). It can be seen that the lattice parameter differences between the calculated and experimental data are less than 1.0%, although the calculated compositions YMn_0.917_TM_0.083_O_3_ are slightly different from the experimental constituents YMn_0.9_TM_0.1_O_3_. The calculated results are in good agreement with the experiments, which proves the reliability of our computations for the doping YMn_0.917_TM_0.083_O_3_ systems (including the hexagonal YMn_0.917_TM_0.083_O_3_ (TM = Ti ∼ Zn) bulks and the YMn_0.917_TM_0.083_O_3_(001)/MgO(001) (TM = Ti, V, Cr and Fe) film configurations).

**Table 1:** Calculated lattice constants and Néel temperatures for the YMn_0.917_TM_0.083_O_3_ compositions compared to experimental values of hexagonal YMn_0.9_TM_0.1_O_3_ (TM = Ti∼Zn) bulks.

TM	Experimental data			Calculated values		
	(*a*, *c*) (Å)	Volume (Å^3^)	*T*_*N*_ (K)	(*a*, *c*) (Å)	Volume (Å^3^)	*T*_*N*_ (K)
Ti	6.141, 11.370	371.35 [6]	55 [6]	6.175, 11.535	380.89	47
V				6.131, 11.433	372.16	63
Cr	6.143, 11.415	373.04 [34]	89 [7], 86 [8]	6.202, 11.539	384.37	99
Mn	6.120, 11.400	369.93 [35]	73 [7], 75 [35]	6.121, 11.408	370.14	70
Fe	6.136, 11.429	372.64 [6]	60 [6], 67 [9]	6.163, 11.460	376.95	62
Co				6.170, 11.492	378.86	63
Ni	42 [11]			6.227, 11.310	379.78	62
Cu	6.152, 11.382	373.05 [11]	59 [12]	6.198, 11.562	384.63	64
Zn	6.179, 11.410	377.25 [12]	70 [5], 60 [13]	6.225, 11.458	384.50	67

**Fig. 2:**
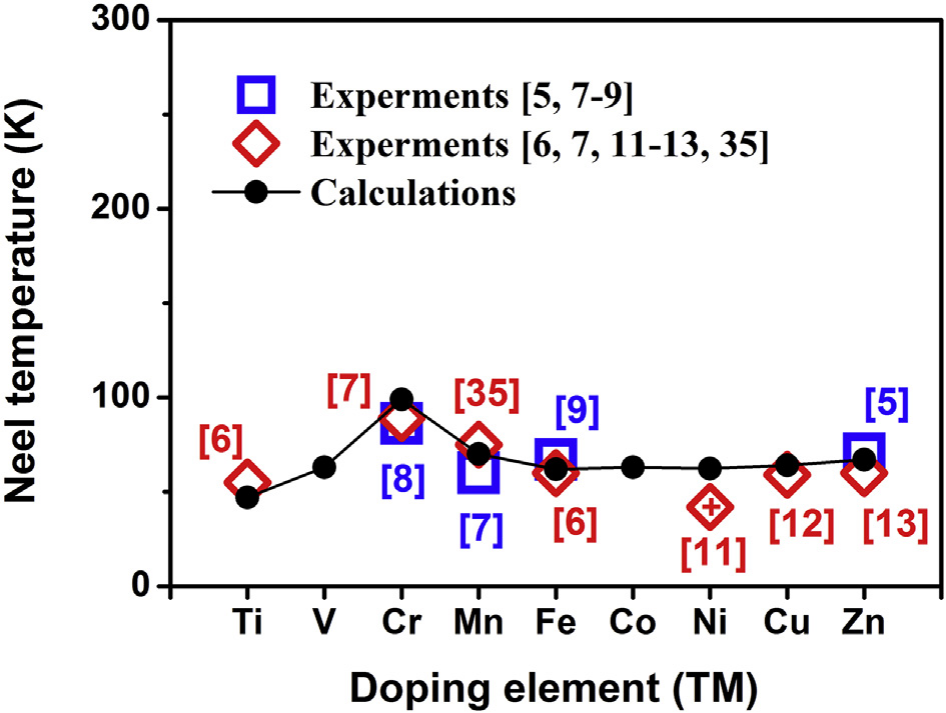
Variation of Néel temperatures with the TM dopant in YMn_0.917_TM_0.083_O_3_ (TM = Ti∼Zn) bulks in comparison with the experimental data. In the figure, the symbol  denotes the reference value from the experimental phase of YMn_0.8_Ni_0.2_O_3_[11].

To study the doping effects on the magnetic properties of hexagonal YMn_0.917_TM_0.083_O_3_ (TM = Ti∼Zn) bulks, the theoretical magnetic transition temperatures (*T*_N_) have been calculated based on the different magnetic structures (such as collinear AFM state, non-collinear magnetic Γ1 state and FM state) and the nearest-neighbor spin-exchange interactions [36]. In all doping systems, the lowest energy state is the Γ1 state, whose lattice parameters are listed in Table 1. The calculated Néel temperatures are also listed. The formula to calculate Néel temperatures will be shown in Section 3.2. Fig. 2 shows that the calculated Néel temperatures varied with doping elements (TM) in the YMn_0.917_TM_0.083_O_3_ bulks, and compared with the reported experimental values of YMn_0.9_TM_0.1_O_3_ (TM = Ti ∼ Zn). It can be seen that the calculated *T*_N_ results clearly depend on doping elements, which agree well with those obtained from experiments. These confirm the sensitivity of magnetic properties to slight perturbations for the hexagonal YMnO_3_ systems. And it is noteworthy that the Cr doping would induce a Néel temperature (*T*_N_) increase higher than the others TM substitutions in the YMn_0.917_TM_0.083_O_3_ bulks.

**Fig. 3:**
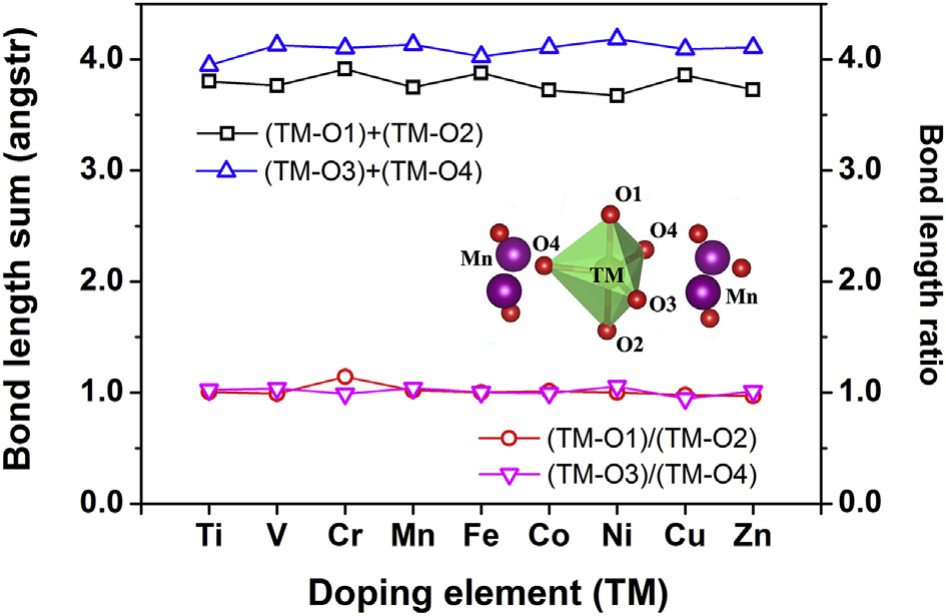
Bond length sums and bond length ratios for TMO_5_ bipyramids in hexagonal YMn_0.917_TM_0.083_O_3_ bulks dependence on doping elements (TM) from Ti to Zn. The inset is the structural representation of TMO_5_ bipyramid.

As is well known, the structural parameters can be varied by suitable substitution and hence one can tune the magnetic property in these types of materials [39]. In the YMn_0.917_TM_0.083_O_3_ compounds, doping at Mn-site would cause the structural distortion of TMO_5_ bipyramid, the magnetic ordering could be driven by the spin-exchange interaction, and the magnetic interaction strength might depend on the bond length and bond angle. Fig. 3 shows that bond length sums {(TM-O1) + (TM-O2), and (TM-O3) + (TM-O4)} and bond length ratios {(TM-O1)/(TM-O2), and (TM-O3)/(TM-O4)} change with doping elements (TM) for TMO_5_ bipyramids in the hexagonal YMn_0.917_TM_0.083_O_3_ bulks. The inset of Fig. 3 displays the TMO_5_ bipyramid structure in which the TM atom is located at the center. It is noted in Fig. 3 that the apical bond length sum {(TM-O1) + (TM-O2)} are always smaller than the planar bond length sum {(TM-O3) + (TM-O4)}, which indicates that TM doping would not change the basic structural characteristics of TMO_5_ bipyramids. It is also noted in Fig. 3 that the bond length ratios {(TM-O1)/(TM-O2), and (TM-O3)/(TM-O4)} are almost same (around the value of 1.0). However, the ratio value of (TM-O1) to (TM-O2) has a notable deviation when Cr is the doping element. This might be one reason why the Néel temperature of YMn_0.917_Cr_0.083_O_3_ exhibits an increase higher than the others YMn_0.917_TM_0.083_O_3_ bulks in Fig. 2.

**Fig. 4:**
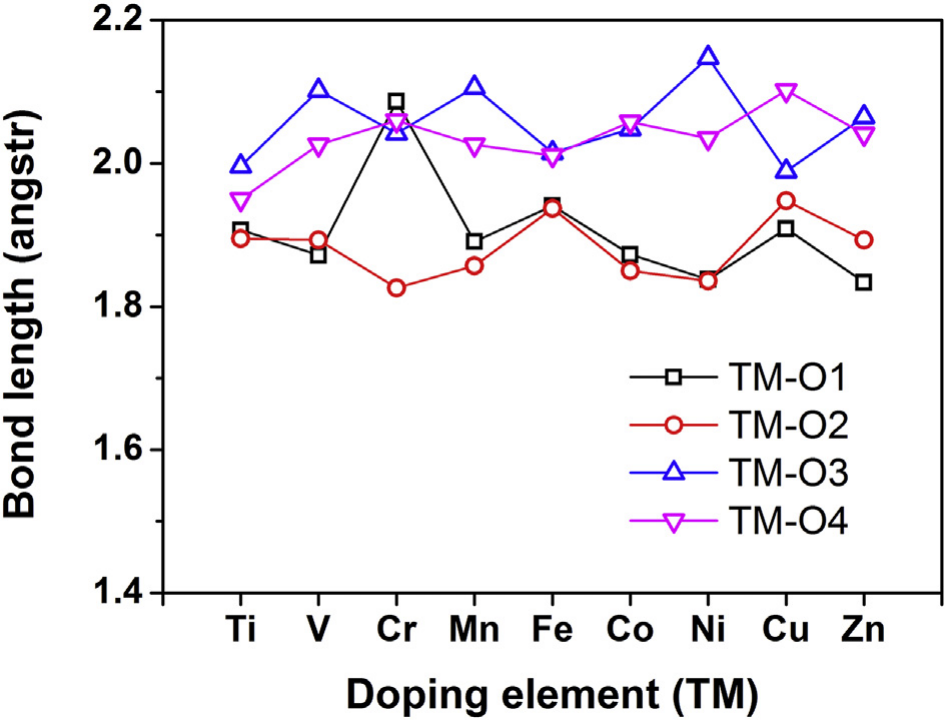
Main bond lengths for TMO_5_ bipyramids in hexagonal YMn_0.917_TM_0.083_O_3_ bulks dependence on doping elements (TM) from Ti to Zn.

**Fig. 5:**
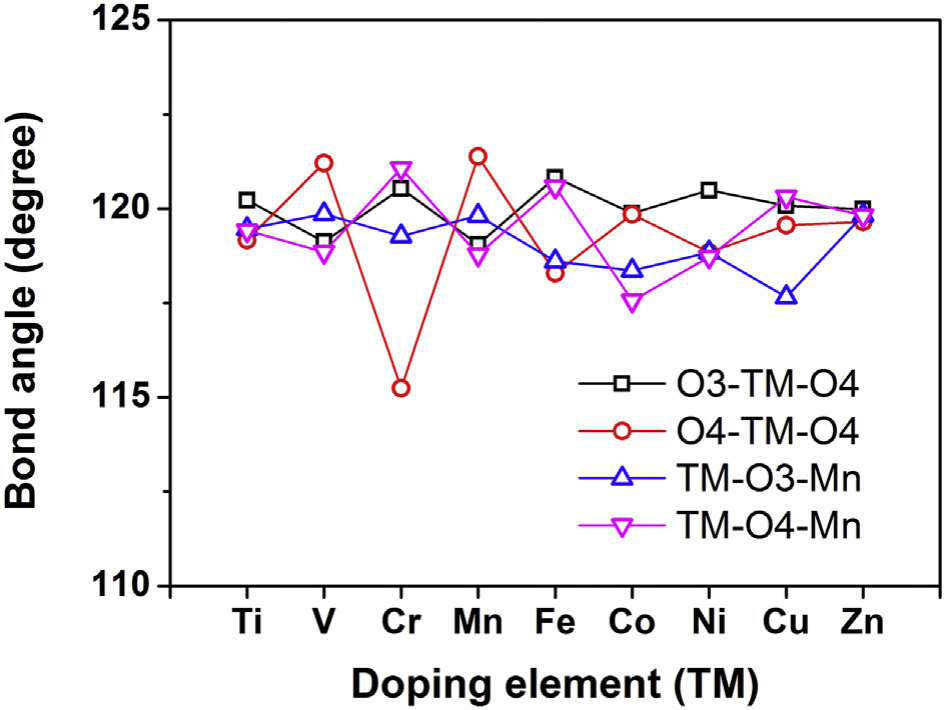
Main bond angles for TMO_5_ bipyramids in hexagonal YMn_0.917_TM_0.083_O_3_ bulks dependence on doping elements (TM) from Ti to Zn.

To further investigate the structural variations of TMO_5_ bipyramids (shown in the inset of Fig. 3), Figs. 4 and 5 respectively demonstrate the bond lengths and bond angles dependence on doping elements (TM) from Ti to Zn. Table 2 lists the calculated main bond lengths and bond angles for TMO_5_ bipyramid in YMn_0.917_TM_0.083_O_3_ bulks, compared to available experimental values in YMn_0.9_TM_0.1_O_3_ (TM = Ti, Mn and Fe) from the previous report [6], which have once again proven that our calculated results are in good agreement with the experimental ones. In Figs. 4 and 5, main bond lengths and bond angles (two structural features) of TMO_5_ bipyramids are respectively changed with different doping elements. It is noteworthy that the variations of TM-O1 distances show a sudden rise when the bond length is Cr-O1 (in Fig. 4), meanwhile the fluctuating curves of O4-TM-O4 angles exhibit a biggest drop at O4-Cr-O4 angle (in Fig. 5), both of which suggest a significant structural distortion occurred in the CrO_5_ bipyramid. When compared between the CrO_5_ and the MnO_5_ bipyramid structures, it is clear in Fig. 3 that the planar bond length sum {(Cr-O3) + (Cr-O4)} is less than the {(Mn-O3) + (Mn-O4)} distance, but the apical bond length sum {(Cr-O1) + (Cr-O2)} is larger than the {(Mn-O1) + (Mn-O2)} distance. As results, the lengthening Cr-O1 distance would decrease the exchange interactions along the [001] direction, and the reduced O4-Cr-O4 angle would cause the bonding strength increase on the Cr-O plane. That is to say, the significant changes of two structural features of CrO_5_ bipyramid would increase the exchange interactions of planar Cr-O bonds and result in a more stable magnetic state, and eventually change the structural and magnetic properties in the hexagonal YMn_0.917_Cr_0.083_O_3_ bulk system (inducing the highest T_N_ peak as shown in Fig. 2).

**Table 2:** Calculated main bond lengths and bond angles for TMO_5_ bipyramid in YMn_0.917_TM_0.083_O_3_ compared to available experimental values [6] in YMn_0.9_TM_0.1_O_3_ (TM = Ti, Mn and Fe).

Geometrical parameter	Ti Exp.	Ti Cal.	Mn Exp.	Mn Cal.	Fe Exp.	Fe Cal.
TM-O1 (Å)	1.940	1.907	1.900	1.891	1.880	1.941
TM-O2 (Å)	1.790	1.895	1.860	1.857	1.820	1.937
TM-O3 (Å)	2.120	1.966	2.082	2.106	2.080	2.015
TM-O4 (Å)	2.023	1.950	2.039	2.026	2.043	2.011
TM-O3-Mn(°)	119.40	119.46	119.24	119.82	119.00	118.61
TM-O4-Mn(°)	119.20	119.42	118.51	118.77	119.10	120.57

### 3.2 For the YMn_0.917_TM_0.083_O_3_(001)/MgO(001) film configurations

By performing DFT calculations, we have also examined variations of electronic structures and magnetic properties of hexagonal YMn_0.917_TM_0.083_O_3_(001)/MgO(001) (TM = Ti, V, Cr and Fe) film configurations. Based on the different Néel spin states (collinear AFM state, non-collinear magnetic Γ1 state and FM state) and the nearest-neighbor spin-exchange interactions, the theoretical magnetic parameters such as transition temperatures (*T*_N_) and Curie-Weiss temperatures ($\Theta_{CW}$) have been calculated [36, 37, 38] to study the doping effects of Ti, V, Cr and Fe atom at Mn site on magnetic properties of YMn_0.917_TM_0.083_O_3_(001)/MgO(001) configurations. The nearest-neighbor spin-exchange coupling *J*_*nn*_ in the hexagonal structure of YMnO_3_ can be calculated as: [36]

$$J_{nn}=\frac{1}{36\left(\sqrt{3}+2\right)}\left(E_{\Gamma_{1}}-E_{AFM}\right)+\frac{1}{216}\left(E_{AFM}-E_{FM}\right)$$

where $E_{AFM}$, $E_{\Gamma_{1}}$ and $E_{FM}$ are respectively total energies for different magnetic configurations (three Néel spin states) as collinear AFM state, non-collinear magnetic Γ1 state and FM state. Then according to L. Capriotti *et al.* [37, 38], the magnetic transition temperature can been estimated as ${T_{N}=-0.3J_{nn}\left(S+\frac{1}{2}\right)}^{2}$ using the nearest-neighbor spin-exchange coupling *J*_*nn*_ and the absolute value of Mn spin *S*. The theoretical Curie-Weiss temperature can be calculated using the expression: ΘCW=(13)⋅Z⋅Jnn⋅S⋅(S+1) [36], where Z is the number of nearest neighbors.

Table 3 lists the calculated values of magnetic parameters of YMn_0.917_TM_0.083_O_3_(001)/MgO(001) (TM = Ti, V, Cr and Fe) configurations. It can be found that Cr doping would also induce a *T*_N_ increase of film configuration similar to the YMn_0.917_Cr_0.083_O_3_ bulk, however, others doping (Ti, V and Fe) would make Néel temperatures reduced to some degree. As we know, the antiferromagnetic ordering temperature of YMnO_3_ is much lower than the absolute value of the Curie-Weiss temperature. Due to the antiferromagnetic interactions, the Curie-Weiss temperatures are negative in the film configurations. It is noted that the variation trends of absolute value of Curie-Weiss temperature $\left|\Theta_{CW}\right|$ are consistent with the Néel temperatures. With the doping elements (Ti, V, Cr and Fe), the calculated effective magnetic moments (*μ*_*eff*_) have decreased slightly when compared to pure YMnO_3_(001)/MgO(001) configuration. Nevertheless, the *μ*_*eff*_ values of Cr, Mn and Fe doping are comparable within the difference (2%). The value of parameter $f=\frac{\left|\Theta_{CW}\right|}{T_{N}}$ is a magnetic frustration factor which can be used as a measure of the spin frustration strength. If the ratio *f* is larger than 10, the spin system should be classified as the one with strong geometrical frustration since the value cannot be explained by the simple mean-field theory [40]. For the hexagonal YMnO_3_ bulk, the calculated Θ_CW_ is −468 K and *T*_N_ is 73 K, then the frustration factor (*f* = 6.41) can be obtained; which is very close to the experimental value (*f* = 6.43) [9]. For the YMn_0.917_TM_0.083_O_3_(001)/MgO(001) configurations, it can be seen in Table 3 that the *f* parameters remain almost the same (the deviations are less than 0.5%) and seem to be independent on the doping elements (TM = Ti, V, Cr and Fe). Because the magnetic interaction strength depends on the extent of orbital overlap and the covalent bond, we next calculated the orbital-resolved density of states (DOS) for further understanding of TM doping effect on electronic structure characteristics of TMO_5_ bipyramids in YMn_0.917_TM_0.083_O_3_(001)/MgO(001) configurations.

**Table 3:** Calculated values of magnetic parameters of YMn_0.917_TM_0.083_O_3_(001)/MgO(001) (TM = Ti, V, Cr and Fe) configurations. Δ*T*_N_ is a difference between the Néel temperature of a doped compound and the pure one. The effective magnetic moment is averaged magnetic moment of TM and Mn in the doped system.

TM	*T*_N_ (K)	Δ*T*_N_ (K)	*Θ*_CW_ (K)	*μ*_eff_ (*μ*_B_)	*f*
Ti	123	-24	-789	4.50	6.415
V	121	-26	-778	4.53	6.429
Cr	161	14	-1031	4.62	6.404
Mn	147 [31]	0	-946	4.70	6.435
Fe	118	-29	-757	4.61	6.415

**Fig. 6:**
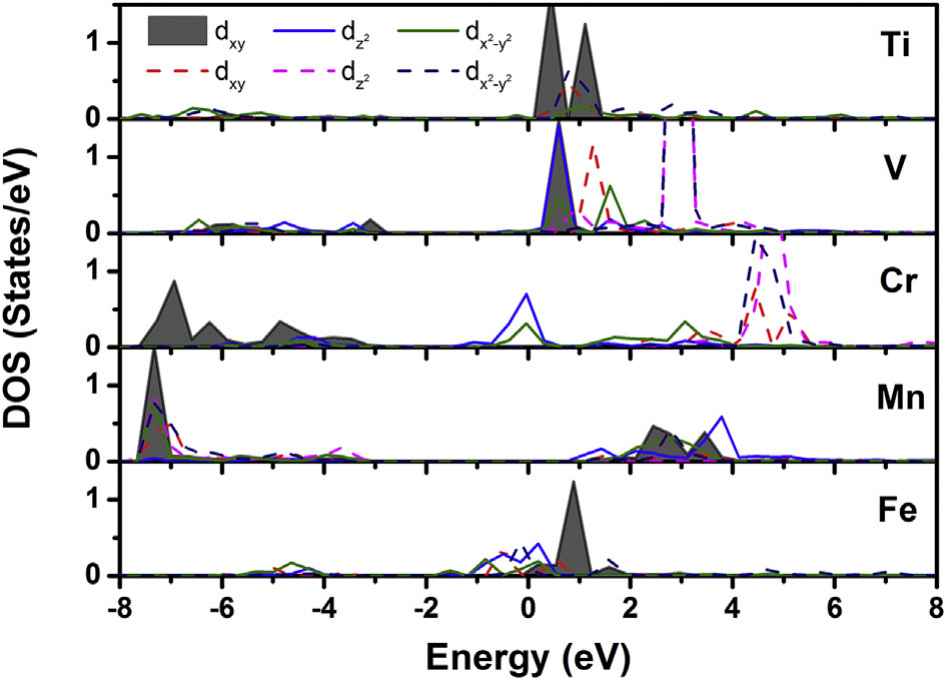
Orbital-resolved density of states of the individual TM (TM = Ti, V, Cr, Mn and Fe) *d* for TMO_5_ bipyramids in YMn_0.917_TM_0.083_O_3_(001)/MgO(001) configurations as a function of energy (*E*-*E*_*F*_), in which *E*_*F*_ is the Fermi energy. Solid and dashed lines denote majority and minority states, respectively.

As we know, the crystal field of hexagonal YMnO_3_ splits the Mn *d* orbitals into two doublets (*e*_1*g*_ and *e*_2*g*_) and a singlet (*a*_1*g*_). In the TMO_5_ bipyramids (shown in the inset of Fig. 3), the *d*_z_^2^ orbitals (*a*_1*g*_) would like to point towards the apical oxygens (O1 and O2) favorable to the bonding {(TM-O1)+ (TM-O2)}, and the *d*_xy_ and *d*_x_^2^_−y_^2^ orbitals (*e*_2*g*_) towards the planar oxygens (O3 and O4) in favor of covalent bonding {(TM-O3)+(TM-O4)}. Fig. 6 shows the orbital-resolved DOSs of the individual TM (TM = Ti, V, Cr, Mn and Fe) *d* for TMO_5_ bipyramids in YMn_0.917_TM_0.083_O_3_(001)/MgO(001) configurations (*d*_yz_ and *d*_zx_ not shown). In the energy range between −7.6 eV and −3.1 eV where O *p* orbitals locate, the TM orbital *d*_z_^2^ DOS's will be responsible for the *d*_z_^2^ (TM)−*p*_z_ (O1 or O2) interaction, and the TM *d*_xy_ (shaded gray in Fig. 6) and *d*_x_^2^_-y_^2^ orbitals will be in charge of hybridization with the in-plane O3(O4) *p*_x_ and *p*_y_. Note that the occupied states of Cr *e*_2*g*_ (*d*_xy_ and *d*_x_^2^_−y_^2^) orbitals illustrate a stronger hybridization trend with O3(O4) (*p*_x_ and *p*_y_) orbitals than the other TM (Ti, V, Mn and Fe) doping's DOS states, and the occupied states of Cr *a*_1*g*_ (*d*_z_^2^) orbitals exhibit a less interaction with O1(O2) (*p*_z_) orbitals than the states of V and Mn orbitals (energy ranging from −7.6 eV to −3.1 eV), all of which suggest that the increase of orbital interactions involved in the planar Cr-O covalent bonds, and the slight decrease of apical Cr-O orbital interactions have both accounted for the increase of magnetic ordering temperature in the YMn_0.917_Cr_0.083_O_3_(001)/MgO(001) configuration.

## 4 Conclusions

In conclusion, the Mn-site doping behaviors of hexagonal YMn_0.917_TM_0.083_O_3_ systems have been systematically studied by using the first-principles method. The calculated Néel temperatures, varied with doping elements in YMn_0.917_TM_0.083_O_3_ bulks, are in agreement with the reported experimental values of YMn_0.9_TM_0.1_O_3_ (TM = Ti ∼ Zn). We have found that the structural changes of TMO_5_ bipyramids would lead to the transformations of magnetic properties. The planar TM-O bonds and O-TM-O angles of TMO_5_ bipyramid are both important structural features for the changes of magnetic ordering temperatures. Then the doping effects of transition metal Ti, V, Cr and Fe at Mn site have also been predicted on the varied magnetic properties of YMn_0.917_TM_0.083_O_3_(001)/MgO(001) film configurations, in which the Cr doping would also induce a *T*_N_ increase higher than the others doping similar to the YMn_0.917_Cr_0.083_O_3_ bulk. By analyses of electronic structures, it can be found that the occupied states of TM *e*_2*g*_ (*d*_xy_ and *d*_x_^2^_−y_^2^) and TM *a*_1*g*_ (*d*_z_^2^) orbitals are simultaneously responsible for the strength of covalent bonds and eventually influence on the magnetic transition temperatures. These insightful results could provide more understanding towards the transition metal doping effect on the structural, electronic and magnetic properties of YMnO_3_ films and the designing of new generation multifunctional devices.

## Declarations

### Author contribution statement

Dong Chen: Analyzed and interpreted the data, Wrote the paper.

Yu-Jia Wang: Conceived and designed the analysis.

Yin-Lian Zhu, Xiu-Liang Ma: Contributed analysis tools or data.

### Funding statement

This work was supported by the National Natural Science Foundation of China(NSFC) (51371176, 51571197 and 51671194), and the Frontier Science Key Programs of the Chinese Academy of Sciences (QYZDJ-SSW-JSC010). Dong Chen was supported by the Special Program for Applied Research on Super Computation of the NSFC-Guangdong Joint Fund (the second phase) under Grant No. U1501501.

### Competing interest statement

The authors declare no conflict of interest.

### Additional information

No additional information is available for this paper.
